# iScore: A ML-Based Scoring Function for De Novo Drug Discovery

**DOI:** 10.1021/acs.jcim.4c02192

**Published:** 2025-03-04

**Authors:** Sayyed Jalil Mahdizadeh, Leif A. Eriksson

**Affiliations:** Department of Chemistry and Molecular Biology, University of Gothenburg, Göteborg 405 30, Sweden

## Abstract

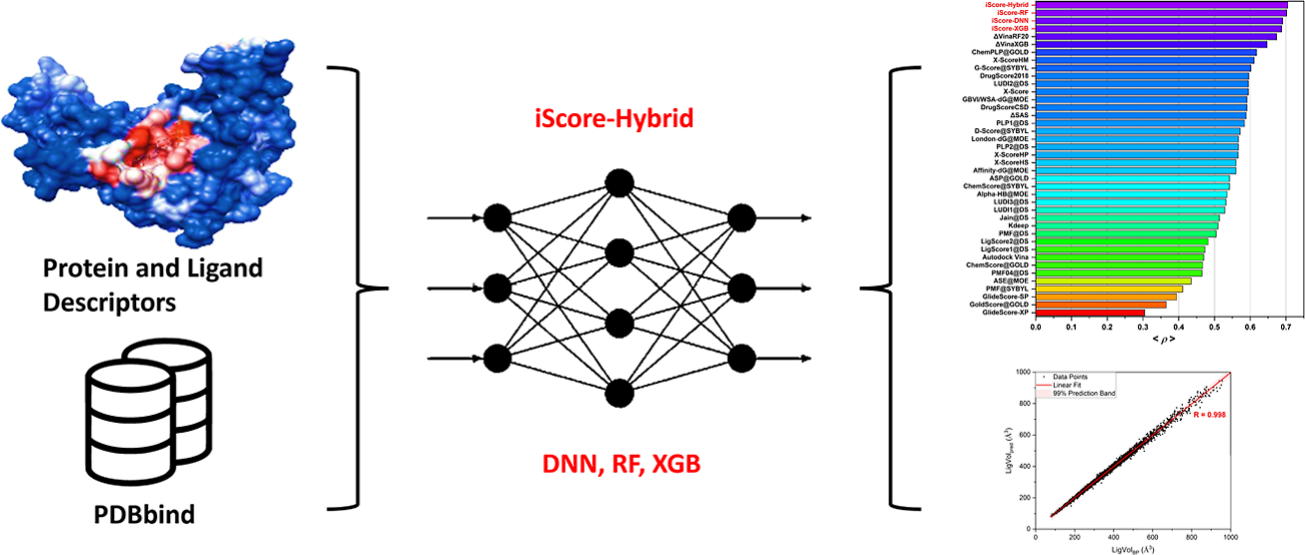

In the quest for accelerating de novo drug discovery, the development of efficient and accurate scoring functions represents a fundamental challenge. This study introduces iScore, a novel machine learning (ML)-based scoring function designed to predict the binding affinity of protein–ligand complexes with remarkable speed and precision. Uniquely, iScore circumvents the conventional reliance on explicit knowledge of protein–ligand interactions and a full picture of atomic contacts, instead leveraging a set of ligand and binding pocket descriptors to directly evaluate binding affinity. This approach enables skipping the inefficient and slow conformational sampling stage, thereby enabling the rapid screening of ultrahuge molecular libraries, a crucial advancement given the practically infinite dimensions of chemical space. iScore was rigorously trained and validated using the PDBbind 2020 refined set, CASF 2016, CSAR NRC-HiQ Set1/2, DUD-E, and target fishing data sets, employing three distinct ML methodologies: Deep neural network (iScore-DNN), random forest (iScore-RF), and eXtreme gradient boosting (iScore-XGB). A hybrid model, iScore-Hybrid, was subsequently developed to incorporate the strengths of these individual base learners. The hybrid model demonstrated a Pearson correlation coefficient (*R*) of 0.78 and a root-mean-square error (RMSE) of 1.23 in cross-validation, outperforming the individual base learners and establishing new benchmarks for scoring power (*R* = 0.814, RMSE = 1.34), ranking power (ρ = 0.705), and screening power (success rate at top 10% = 73.7%). Moreover, iScore-Hybrid demonstrated great performance in the target fishing benchmarking study.

## 1 Introduction

Molecular docking is undoubtedly the most widely used technique in structure-based computer-aided drug discovery that aims to predict the binding mode and binding affinity of small organic molecules toward a target protein.^1^ The performance (speed and accuracy) of a molecular docking program strongly depends on its two main components, sampling and scoring.^2^ Sampling refers to a search algorithm that evaluates a finite number of ligand conformations within and around the binding site of a target protein to elucidate the ligand binding mode. Scoring refers to a class of computational methods, called scoring functions, that are formulated to predict the binding affinity of each ligand conformation within the protein binding site.^3^ The performance of a scoring function can be determined by three evaluation metrics:^4^ “scoring power” that indicates the degree of correlation in the predicted versus experimentally determined binding affinity values, “ranking power” that is the capability of scoring function for accurately ranking a given set of active ligands with respect to their predicted binding affinity values, toward a particular protein target, and “screening power” that refers to the ability of scoring function to identify the true ligand with the highest affinity against a given protein target among a set of random decoy molecules. While the scoring part of a typical molecular docking calculation is relatively fast, the sampling part is time-consuming, computationally expensive, and inefficient.^5^ Therefore, in the traditional molecular docking and virtual screening, the calculation time and cost scale exponentially with an increasing number and degree of freedom of molecules under evaluation. On the other hand, the size of available molecular databases for virtual screening remains extremely limited, ranging from several million to a few billion molecules. This represents only a tiny fraction of the actual chemical space, which is estimated to contain up to ∼10^60^ feasible drug-like molecules.^6^

Traditional scoring functions can be categorized in three main classes based on the way they are formulated: force-field based, empirical, and knowledge-based scoring functions.^7^ Despite significant improvements in the past decade, several recent studies clearly show that the performance of traditional scoring functions is quite limited in both scoring power and ranking power aspects.^8^ On the other hand, the most successful scoring approaches, such as free-energy perturbation (FEP) techniques,^9^ are very sensitive to the force field selection and ligand parametrization. Moreover, the widespread application of FEP methods has been significantly constrained by their high computational demands, even for small molecular libraries. However, recent breakthroughs in machine learning (ML) algorithms and big data mining, coupled with the exponential growth of computing power, have paved the way for promising applications of ML-driven approaches in the development of novel scoring functions.^10^ ML-based scoring functions have demonstrated remarkable performance in various benchmarking studies.^11^ They are also typically several orders of magnitude faster than traditional scoring functions. Stepniewska-Dziubinska et al.^12^ developed a deep neural network (DNN), Pafnucy, to estimate the binding affinity of ligand–receptor complexes. The model represents the complex using a 3D grid and applies 3D convolution to generate a feature map from this representation, treating atoms from both protein and ligand equivalently. Their model was tested on the CASF-2013 and CASF-2016 “scoring power” benchmark showing good performance with a Pearson correlation coefficient/root mean squared error (RMSE) of 0.70/1.62 and 0.78/1.42, respectively. Zheng et al.^13^ employed a deep convolutional neural network model called OnionNet for protein–ligand binding affinity prediction. This model utilizes rotation-free, element-pair-specific contact features between ligand and protein atoms, categorized into different distance ranges to capture both local and nonlocal interaction information. The predictive performance of OnionNet was evaluated using the CASF-2013 and CASF-2016 benchmarks, achieving strong results with Pearson correlation coefficients/RMSE of 0.78/1.50 and 0.81/1.28, respectively. Using 3D convolutional neural network (3D-CNN) architecture, Li et al.^14^ developed a model called DeepAtom. This model automatically extracts binding-related atomic interaction patterns from the voxelized structure of the complex and was used for the binding affinity prediction. DeepAtom achieved a Pearson correlation coefficient and RMSE values of 0.81 and 1.32 on the CASF-2016 benchmark, respectively. Wang et al.^15^ developed a deep learning approach, named DeepDTAF, to predict the protein–ligand binding affinity. DeepDTAF was constructed by integrating local and global contextual features of the protein and ligand encoded by one-hot encoding and integer encoding, respectively. The prediction performance of DeepDTAF was evaluated on the CASF-2016 benchmark showing Pearson correlation coefficient and RMSE values of 0.75 and 1.44, respectively. Using DNN and atom–atom pairwise interactions, Moon et al.^16^ developed a model, PIGNet, for protein–ligand binding affinity predictions. The model generalization of PIGNet was further improved by augmenting the training data with a broader range of binding poses and ligands. The evaluation of the model on the CASF-2016 test set demonstrated a Pearson correlation coefficient of 0.76. Li et al.^17^ developed a multiobjective neural network (MONN) to predict both noncovalent interactions and binding affinities between compounds and proteins. They compiled a benchmark data set containing noncovalent intermolecular interactions for more than 10,000 compound–protein pairs and systematically evaluated the interpretability of neural attentions in existing models. MONN was evaluated on the BindingDB data set^18^ achieving excellent Pearson correlation coefficient and RMSE values of 0.86 and 0.76, respectively. For further details on studies employing various ML techniques for protein–ligand binding affinity prediction, we refer to the review paper by Zhang et al.^19^

Regardless of whether they are classified as classical or modern, all scoring functions developed for receptor–ligand binding affinity prediction face a common challenge: the need for a clear and explicit understanding of protein–ligand interactions (e.g., hydrogen bonds, polar and hydrophobic interactions, and van der Waals contacts). This necessitates a slow and computationally expensive sampling process before scoring calculations can be performed. Consequently, while modern ML-based scoring functions are significantly faster and more accurate, their integration into molecular docking pipelines still struggles to overcome these limitations due to the inherent “bottleneck” in the sampling stage.

In this study, we introduce a novel ML-based scoring function (iScore) that quickly and precisely predicts the binding affinity of protein–ligand complexes without the need for knowledge of explicit intermolecular interactions. Instead, iScore predicts the protein–ligand binding affinity based on a combination set composed of the ligand and binding pocket descriptors. Therefore, the sampling stage can be avoided, which leads to massive savings in time and resources. On the other hand, since iScore architecture is independent of explicit intermolecular interactions, it can be employed to score and rank huge libraries of de novo small molecules against a protein target of interest, which greatly assists researchers in evaluating “unseen” regions of chemical space. iScore has been trained on the PDBbind 2020^20^ refined set using three different ML approaches: DNN (iScore-DNN), Random Forest (RF) (iScore-RF), and eXtreme gradient boosting (iScore-XGB). Furthermore, a hybrid scoring function (iScore-Hybrid) has been developed by combining and taking advantage of these three base learners. The scoring power, ranking power, screening power, and target fishing performances of iScore have been extensively tested and compared with other traditional and ML-based scoring functions using various test sets: PDBbind 2016 core set (Comparative Assessment of Scoring Functions, CASF-2016),^4^ two data sets from the Community Structure–Activity Resource (CSAR NRC-HiQ Set1 and CSAR NRC-HiQ Set2),^21^ Database of Useful Decoys-Enhanced (DUD-E),^22^ and target fishing data set.^23^ It is important to emphasize that iScore is not a traditional scoring function designed to assess binding poses or ligand conformations. Instead, it relies on the complementarity between molecular descriptors of ligands and binding pocket features, directly predicting binding affinity without the need for a 3D conformational search or explicit calculations of binding energy components. We believe that iScore paves the way for a new era in de novo drug discovery and pharmaceutical research.

## 2 Materials and Methods

### 2.1 Data Set Preparation

The iScore models have been trained using the PDBbind 2020 refined set as a training data set.^20^ The PDBbind 2020 refined set (consisting of 5316 protein–ligand complexes along with the associated experimental affinity data) is a cherry-picked subset of the PDBbind 2020 general set (over 23,496 complexes) by selecting the complexes with no obvious structural issues or steric clashes, crystal resolution <2.5 Å, R-factor <0.25, noncovalently bound ligand, and affinity data reported in either *K*_d_ or *K*_i_ form in the range of 10 mM to 1 pM. A full description of the criteria used for defining the PDBbind refined set can be found in the original paper.^4^ The PDBbind 2016 core set, the first test set in our study and in the CASF-2016 benchmarking, was selected from the PDBbind refined set by applying even strict criteria as follows: (1) the PDBbind refined set was subjected to a sequence similarity clustering with a similarity cutoff of 90% and only the clusters containing more than 5 members were considered, (2) five representative complexes were selected for each remaining cluster based on their affinity data, those with the highest and lowest affinities with at least 100-fold difference, and three additional (intermediate) complexes, (3) the ligands should not be identical or stereoisomers throughout the PDBbind core set, and (4) the electron density map and the ligand binding pose in each complex should be of high quality. This resulted in 285 protein–ligand complexes clustered into 57 clusters in the PDBbind 2016 core set. The two other test data sets used in this study are CSAR NRC-HiQ Set1 and CSAR NRC-HiQ Set2 containing 176 and 167 high-quality protein–ligand complexes, respectively.

Prior to database preparation, the overlapping complexes between the PDBbind 2020 refined set and the PDBbind 2016 core set were removed from the training set. In addition, the overlapping complexes between the PDBbind 2020 refined set and CSAR NRC-HiQ Set1/Set2 were removed from these test sets. The crystal structures were subsequently prepared using the PrepWizard in the Schrödinger 2023-2 program package (https://www.schrodinger.com/). Hydrogen atoms were incorporated, and missing side chain atoms were added by using Prime. After fixing the potential structural defects, water molecules were removed from the complexes and the protonation states of ionizable residues were determined at pH = 7.0 using PROPKA.^24^ The correct protonation states of the ligand molecules were also determined at pH = 7.0 using Epik.^25^ The prepared complexes were further refined using the OPLS4 force field^26^ in a restrained minimization procedure with an RMSD threshold of 0.3 Å for all heavy atoms. The complexes that failed during the preparation stage were discarded. The final prepared data sets contain 4898 (PDBbind 2020 refined set), 285 (PDBbind 2016 core set), 68 (CSAR NRC-HiQ Set1), and 75 (CSAR NRC-HiQ Set2) complexes. The PDB codes for each protein in the data sets are listed in Table S1.

The DUD-E^22^ and target fishing^23^ data sets were prepared following the same protocol as described above. DUD-E consists of 102 target proteins with an average of 224 active compounds per target and 50 decoys for each active, resulting in 22,886 actives and 1,144,300 decoys, respectively. For each target, the decoys share similar 1D physicochemical properties with known actives, such as molecular weight and LogP, but differ in 2D topology descriptors. The target fishing data set consists of 122 drugs with 6348 known target proteins from 1860 unique PDB-ids compiled from BindingDB^18^ (http://www.bindingdb.org) and DrugBank^27^ (http://www.drugbank.ca).

### 2.2 Descriptor Calculations

#### 2.2.1 Ligand Descriptors

The 3D structures of the ligand molecules were converted to the corresponding canonical simplified molecular-input line-entry system (SMILES)^28^ strings and subsequently a series of 81 1D/2D molecular descriptors were calculated using the RDKit library (https://www.rdkit.org) in Python such as logarithm of the partition coefficient (MolLogP), molecular refractivity (MolMR), exact molecular weight (ExactMolWt), number of heavy atoms (HeavyAtomCount), number of hydrogen-bond acceptors (NumHAcceptors), number of hydrogen-bond donors (NumHDonors), and number of rotatable bonds (NumRotatableBonds). A full list of the molecular descriptors used in this study is presented in Table S2. Figure S1 shows the histogram distribution of some molecular descriptors of the ligands in the training set along with the logarithmic form of the experimental binding affinity values (*pK*_d_).

#### 2.2.2 Binding Pocket Descriptors

The FPocket^29^ tool was employed to calculate 41 descriptors of the protein binding pocket such as the pocket volume (pock_vol), number of alpha spheres (nb_AS), mean alpha sphere radius (mean_as_ray), mean alpha sphere solvent accessibility (mean_as_solv_acc), polarity score (polarity_score), hydrophobicity score (hydrophobicity_score), charge score (charge_score), volume score (volume_score), and amino acid composition. The protein binding pocket was explicitly defined by all atoms situated at a certain cutoff distance from the ligand molecule (3–7 Å). The initial assessments showed that a cutoff distance of 5 Å resulted in the best training and binding affinity prediction performance. A full list of the binding pocket descriptors is presented in Table S2. Figure S2 shows the histogram distribution of some descriptors of the binding pocket in the training data set. Furthermore, FPocket suggests an intuitive estimation for the volume of potential ligands (LigVol_BP_) which was used in this study as a descriptor in training of the scoring models and as a key feature in training of the ultra-fast screening (UFS) model which was used to improve the screening power performance by further filtering false positives (Section 2.4.3).

### 2.3 ML Algorithms

iScore has been trained using three different ML approaches: DNN,^30^ RF,^31^ and XGB.^32^ In this study, the hyperparameters of iScore-RF and iScore-XGB models were automatically tuned with the Bayesian optimization^33^ technique implemented in the Scikit-learn^34^ version 1.0.2, while Keras version 2.4.3 (https://keras.io)^35^ was employed for hyperparameter optimization of the iScore-DNN model. A 3-fold cross-validation was used to evaluate various hyperparameter combinations, and RMSE was utilized as the object function. The maximum iteration was set to 200.

#### 2.3.1 Deep Neural Network

The Keras package version 2.4.3 in Python 3 was employed to build the iScore-DNN model. The DNN model consists of six layers: an input layer (81 + 41 neural nodes), four hidden layers with 350, 250, 150, and 50 neural nodes, and an output single node layer. The RELU^36^ activation function was used for all layers except the output layer where a LINEAR activation function was employed. The loss function and evaluation metric were set to mean-absolute error and mean-squared error, respectively. An inverse-time-decay scheduler with an initial learning rate of 0.001, a decay rate of 0.3, and a decay step of 8000 was used to properly lower the learning rate during the training process with the Adam optimizer and 100 epochs. iScore-DNN was trained thorough 10 × 10-fold cross validation (XV) with random data shuffling in each XV loop. The final output was an average value of over 100 DNN XV models.

#### 2.3.2 Random Forest

The Scikit-learn package version 1.0.2 in Python 3 was used to build the iScore-RF model. The RF consisted of 200 decision trees (n_estimators) with min_samples_split (minimum number of samples required to split an internal node) = 2, min_samples_leaf (minimum number of samples required to be at a leaf node) = 1, and max_features (number of features to consider when looking for the best split) = “auto”. The criterion was set to mean-squared error and the estimators were allowed to expand until all leaves were pure. iScore-RF was trained thorough 10 × 10-fold XV with a random data shuffling in each XV loop. The final output was an average value over 100 RF XV models.

#### 2.3.3 eXtreme Gradient Boosting

The XGBoost package version 1.5.2 in Python 3 was used to build the iScore-XGB model. The hyperparameters of the XBG trainer are n_estimators (number of estimators) = 1000, learning_rate = 0.01, subsample (Subsample ratio of the training instances prior to growing estimators) = 0.7, colsample_bytree (subsample ratio of columns when constructing each tree) = 1.0, max_depth (maximum depth of an estimator) = 8, and objective (regression type) = “reg/squarederror”. iScore-XGB was trained thorough 10 × 10-fold XV with a random data shuffling in each XV loop. The final output was an average value over 100 XGB XV models.

#### 2.3.4 Hybrid Model

A hybrid scoring function (iScore-Hybrid) was subsequently developed by combining the iScore-DNN, iScore-RF, and iScore-XGB models. For this purpose, the average predicted affinity values over 100 XV of each model (iScore-DNN, iScore-RF, and iScore-XGB) along with experimental affinity data were fed into a DNN trainer. The iScore-Hybrid model consists of six layers: an input layer, four hidden layers with 100, 50, 10, and 50 neural nodes, and an output single node layer. The RELU activation function was used for all layers except the output layer, where a LINEAR activation function was employed. The loss function and evaluation metric were set to mean-absolute error and mean-squared error, respectively. An inverse-time-decay scheduler with an initial learning rate of 0.001, decay rate of 0.3, and decay steps of 8000 was used to properly lower the learning rate during the training process with the Adam optimizer and 100 epochs. iScore-Hybrid was trained through 10 × 10-fold XV with a random data shuffling in each XV loop. The final output was an average value over 100 DNN XV models.

### 2.4 Evaluation Metrics

#### 2.4.1 Scoring Power

“Scoring power” indicates the degree of correlation between the predicted and experimentally determined binding affinity values. Hence, the Pearson correlation coefficient (*R*) was computed as a quantitative indicator of scoring power (eq 1).^4^ The RMSE of the regression was considered as an additional indicator (eq 2).^4^
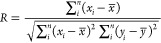
1
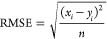
2where *x*_*i*_, *y*_*i*_, *x̅*, and *y̅* are the estimated and experimental binding affinity of the *i*th complex and the corresponding average values, respectively. The summation upper limit (*n*) is the total number of complexes, i.e., 4898 (PDBbind 2020 refined set), 285 (PDBbind 2016 core set), 68 (CSAR NRC-HiQ Set1), and 75 (CSAR NRC-HiQ Set2).

#### 2.4.2 Ranking Power

“Ranking power” refers to the capability of the scoring function in ranking a given set of active ligands, with respect to their predicted binding affinity values, toward a particular protein target. The PDBbind 2016 core set contains 285 protein–ligand complexes clustered into 57 clusters. Each cluster contains a particular target receptor and 5 different active binders where the difference between binding affinities of the strongest and weakest binders is at least 100-fold. Figure S3 shows a boxplot of experimental binding affinity values for each of the 57 clusters in the PDBbind 2016 core set. The Spearman ranking correlation (ρ, eq 3)^4^ was used as an indicator of the ranking power (as CASF-2016 benchmarking) since in contrast to scoring power, ranking power does not request a linear correlation between experimental and predicted binding affinity values.
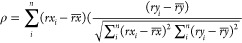
3where *rx*_*i*_, *ry*_*i*_, , and  are the rank of estimated and experimental binding affinity of the *i*th complex and the corresponding average values, respectively. The summation upper limit (*n*) is the total number of samples in each cluster, i.e., five in this case. The average Spearman ranking correlation, ⟨ρ⟩, was subsequently calculated over all 57 target proteins in the PDBbind 2016 core set.

#### 2.4.3 Screening Power

“Screening power” indicates the ability of a scoring function to identify the true binder with the highest affinity for a given protein target among a set of random decoy molecules. The first quantitative reference metric of screening power is the success rate of identifying the ligand with highest affinity against each of the 57 target receptors in the PDBbind 2016 core set, among the 1%, 5%, and 10% top candidates. The second indicator is the success rate of identifying all binders with experimental binding affinity values less than 10 mM (*pK*_d_ ≥ 2), 10 μM (*pK*_d_ ≥ 5), 1 μM (*pK*_d_ ≥ 6), 0.1 μM (*pK*_d_ ≥ 7), 0.01 μM (*pK*_d_ ≥ 8), and 1 nM (*pK*_d_ ≥ 9), among the 1%, 2%, 3%, 5%, and 10% top candidates over all 285 complexes. There are 285, 213, 167, 117, 75, and 39 binders with experimental binding affinity values less than 10 mM, 10 μM, 1 μM, 0.1 μM, 0.01 μM, and 1 nM in the PDBbind 2016 core set, respectively.

**Figure 1:**
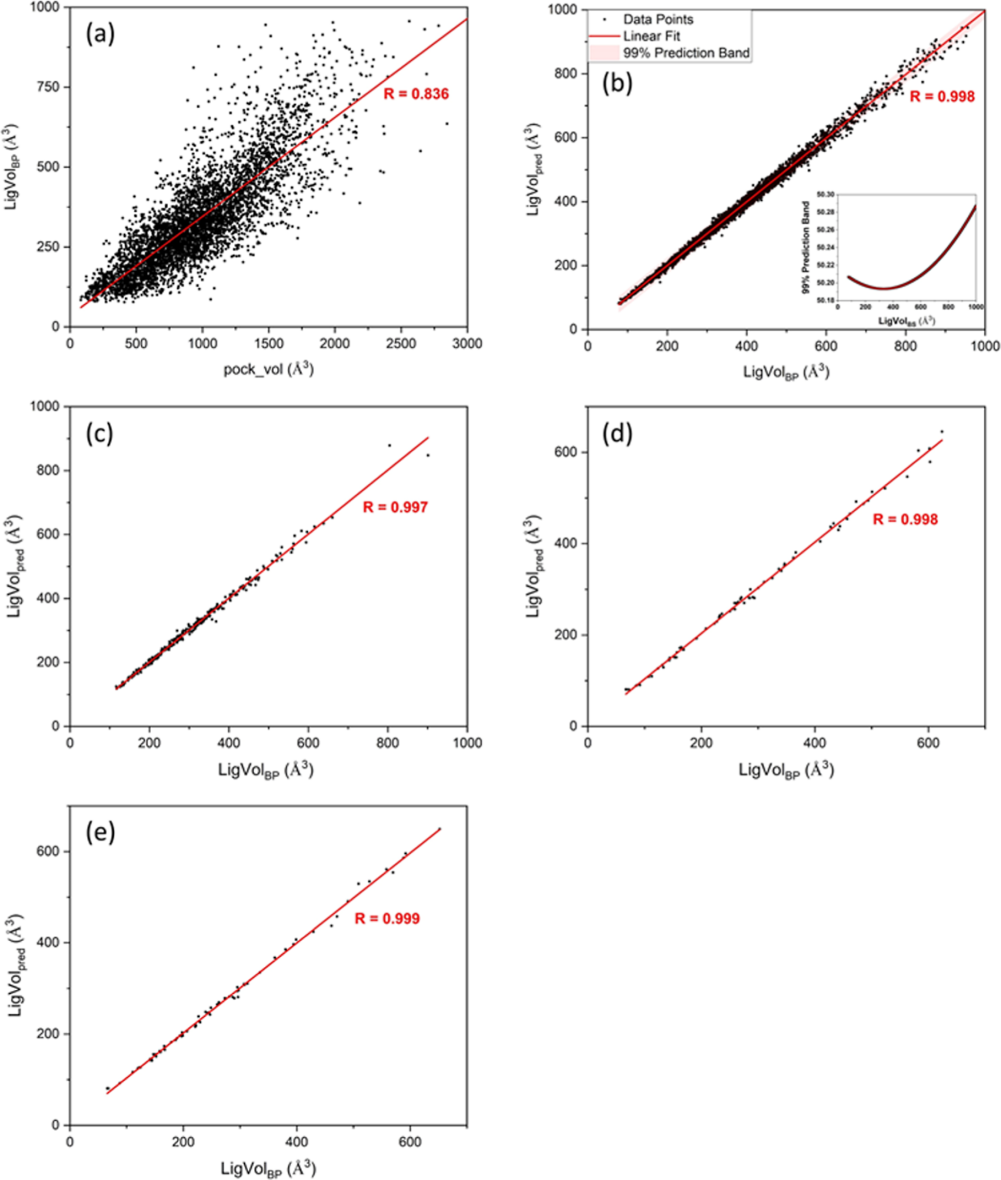
(a) Strong correlation between the LigVol_BP_ and pock_vol. (b) Correlation between LigVol_pred_ estimated from 2D molecular descriptors and LigVol_BP_, with the 99% prediction band region calculated upon 3 × 10-fold XV on the training set. The performance of the RF volume predictor on the (c) PDBbind 2016 core set, (d) CSAR NRC-HiQ Set1, and (e) CSAR NRC-HiQ Set1. The Pearson correlation coefficients (*R*) are shown in each graph.

The screening power performance of iScore models was further improved by an UFS stage prior to the binding affinity prediction. In the UFS stage, the ligands that volumetrically do not match with a given receptor’s binding pocket (too big or too small) will be filtered out. One of the features that the FPocket tool predicts, after receptor binding pocket evaluation, is an intuitive estimation of the volume of potential binders (LigVol_BP_) that strongly correlates with the receptor binding pocket volume (pock_vol) (Figure 1a). Hence, an RF-based regression model was trained to calculate LigVol_BP_ based on 81 1D/2D molecular descriptors (LigVol_pred_) listed in Table S2. From the correlation graph, the 99% prediction band (Figure 1b) was calculated upon 3 × 10-fold XV and used in the UFS stage, so that only the ligands with predicted volumes (LigVol_pred_) within the 99% prediction band from the LigVol_BP_ value were allowed to pass to the scoring stage. The trained RF volume predictor has been tested on three test sets used in this study (PDBbind 2016 core set, CSAR NRC-HiQ Set1 and Set2) and the results showed very close to perfect correlation (Figure 1c–e).

## 3 Results

### 3.1 Training

**Figure 2:**
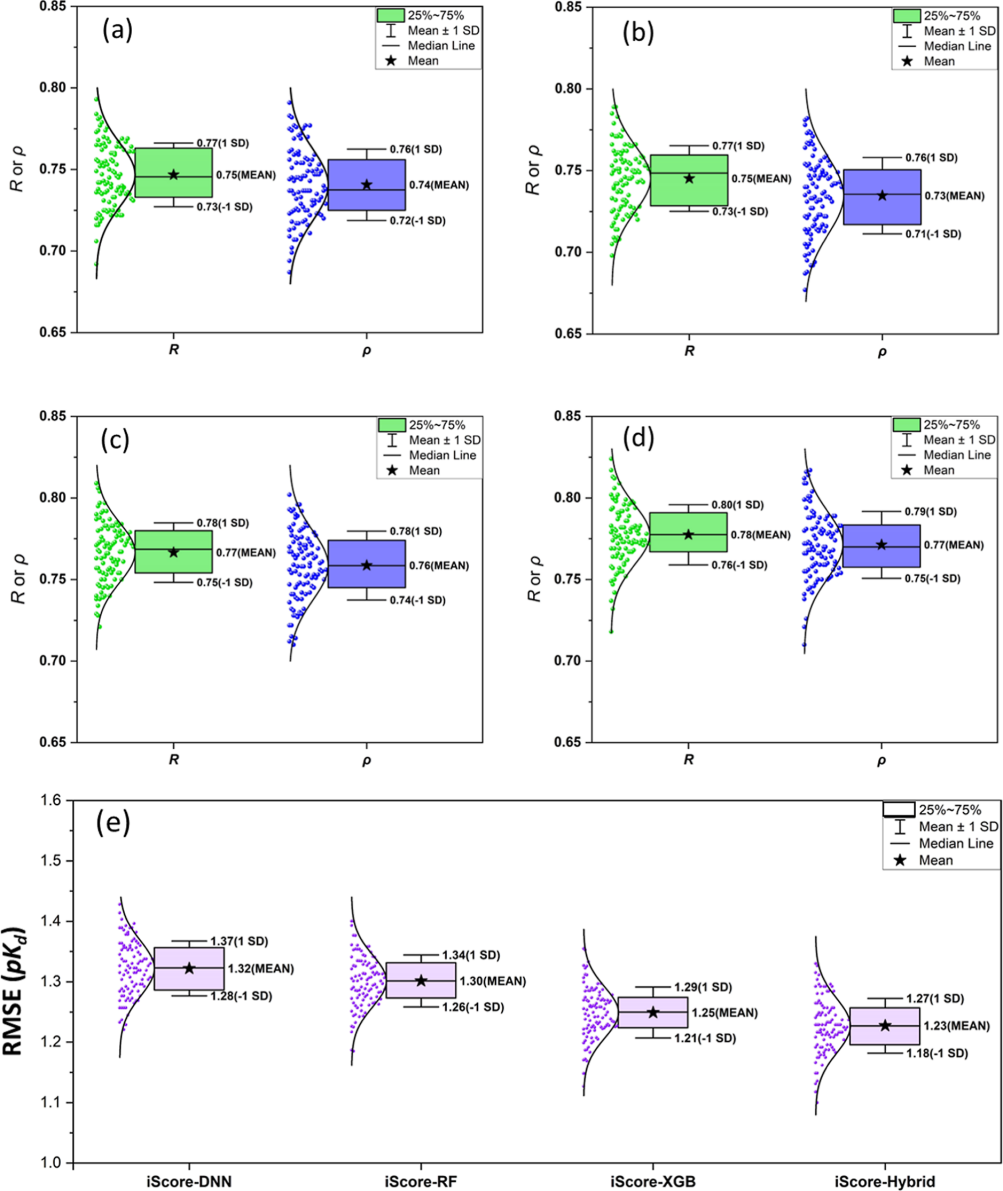
Boxplot presentation and distribution of Pearson (*R*) and Spearman (ρ) correlation confections along with the mean, median, and SD values for (a) iScore-DNN, (b) iScore-RF, (c) iScore-XGB, and (d) iScore-Hybrid upon 10 × 10-fold XV training campaign. (e) RMSE statistics for the base learners along with the hybrid model.

Figure 2a–d shows the 25%–75% boxplot presentation and distribution of Pearson (*R*) and Spearman (ρ) correlation confections along with the mean, median, and standard deviation (SD) values for the models trained with different ML algorithms (base learners) and the hybrid model, upon 10 × 10-fold XV training campaign. The mean Pearson coefficients are 0.75, 0.75, 0.77, and 0.78 for the iScore-DNN, iScore-RF, iScore-XGB, and iScore-Hybrid models, respectively. The SD profile of the Pearson coefficient is similar for all models and lies in the range of 0.01–0.02. As these figures indicate, the mean Spearman coefficients (ρ) are slightly lower than the mean Pearson coefficients, but the same SD values have been observed. Figure 2e shows the RMSE statistics for three base learners along with the hybrid model. The mean RMSE values are 1.32, 1.30, 1.25, and 1.23 for the iScore-DNN, iScore-RF, iScore-XGB, and iScore-Hybrid models, respectively. The SD profiles of the RMSE metric are similar and cover the range of 0.04–0.05. The results clearly show that iScore-Hybrid outperforms the base learners with higher mean Pearson and Spearman correlation coefficients and lower mean RMSE values in the cross-validation training campaign. The scatter plots comparing the predicted to experimental values for the internal 10-fold cross validation are shown in Figure S4a–d. The 99% prediction band (pink area), Pearson correlation coefficient (*R*), and RMSE values are shown for each correlation.

**Figure 3:**
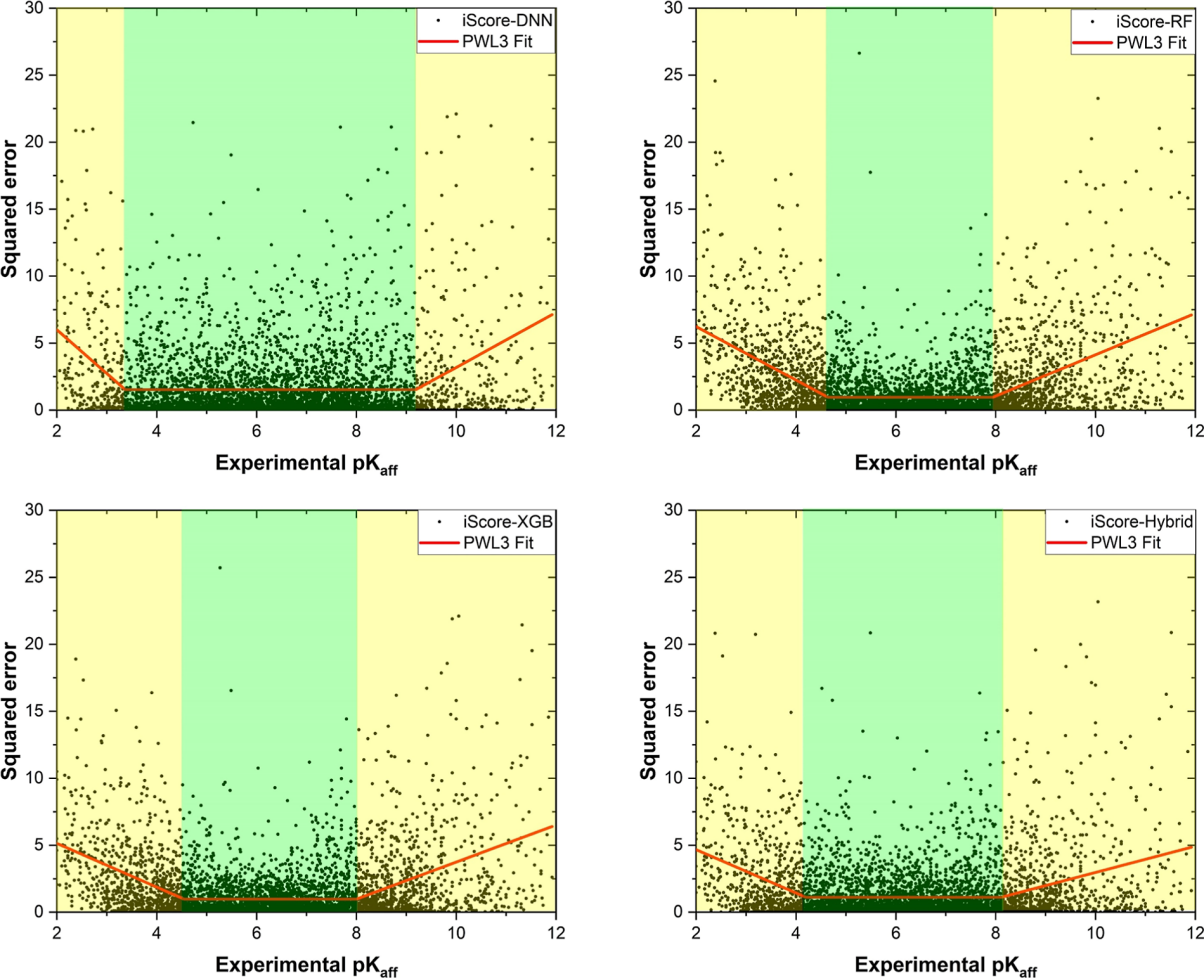
Squared error versus experimental *pK*_d_ obtained from each model upon 10 × 10-fold XV training campaign. The piecewise linear function with three segments (PWL3) fitted into the data is shown in red. The green and yellow dashed areas illustrate the trust zone and nonzero-slope regions, respectively.

To further understand the better performance of the iScore-Hybrid over all base learners, the plots of the squared error (squared difference between the experimental and the predicted *pK*_d_) versus the experimental *pK*_d_ (Figure 3) associated with each model have been deeply explored. As Figure 3 illustrates, there are three distinct regions that were quantitatively distinguished after fitting the data into a piecewise linear function with three segments (PWL3). The first region (green dashed area) is the trust zone in the midrange *pK*_d_ spectrum where the PWL3 function forms a horizontal line indicating the most reliable range of *pK*_d_ that the model can predict at the maximum accuracy (minimum error). The other two regions are at the respective ends of the experimental *pK*_d_ (yellow dashed areas) where the PWL3 function forms nonzero-slope lines. One can elucidate the overall performance of the models by comparing three determinative factors: the trust zone’s length (the bigger the better) and height (the lower the better) and the absolute slope of the lines in the nonzero-slope regions (the lower the better). The maximum trust-zone length is 5.82 [3.37, 9.19] which belongs to the iScore-DNN, while the corresponding values are 3.30 [4.64, 7.94], 3.45 [4.55, 8.00], and 3.91 [4.20, 8.11] for iScore-RF, iScore-XGB, and iScore-Hybrid, respectively. On the other hand, iScore-DNN has the maximum trust-zone height of 1.53 followed by 1.12 (iScore-Hybrid), 0.99 (iScore-XGB), and 0.97 (iScore-RF). Moreover, the minimum absolute slopes of the lines in the nonzero-slope regions belong to iScore-Hybrid which are 1.60 and 0.98 at the low- and high-affinity limits, respectively. The corresponding values are (1.63 and 1.38), (2.00 and 1.54), and (3.27 and 2.05), for iScore-XGB, iScore-RF, and iScore-DNN, respectively. Therefore, in the context of the trust-zone length, iScore-Hybrid showed a better performance than the two base learners iScore-RF and iScore-GXB. In the context of the trust-zone height, iScore-Hybrid outperforms the iScore-DNN by a considerable margin. Interestingly, iScore-Hybrid furthermore showed the best performance at both low- and high-affinity limits, where the base learners suffer from the lower prediction accuracy.

### 3.2 Benchmarks

The scoring, ranking, and screening power performances of the iScore models have been extensively tested and compared to other traditional and ML-based scoring functions on three different test sets: the PDBbind 2016 core set (scoring, ranking, and screening power), CSAR NRC-HiQ Set1 and Set2 (scoring performance), and DUD-E (screening power). Moreover, the target fishing capability of the models was evaluated and compared to various scoring functions.

#### 3.2.1 Scoring Power

**Figure 4:**
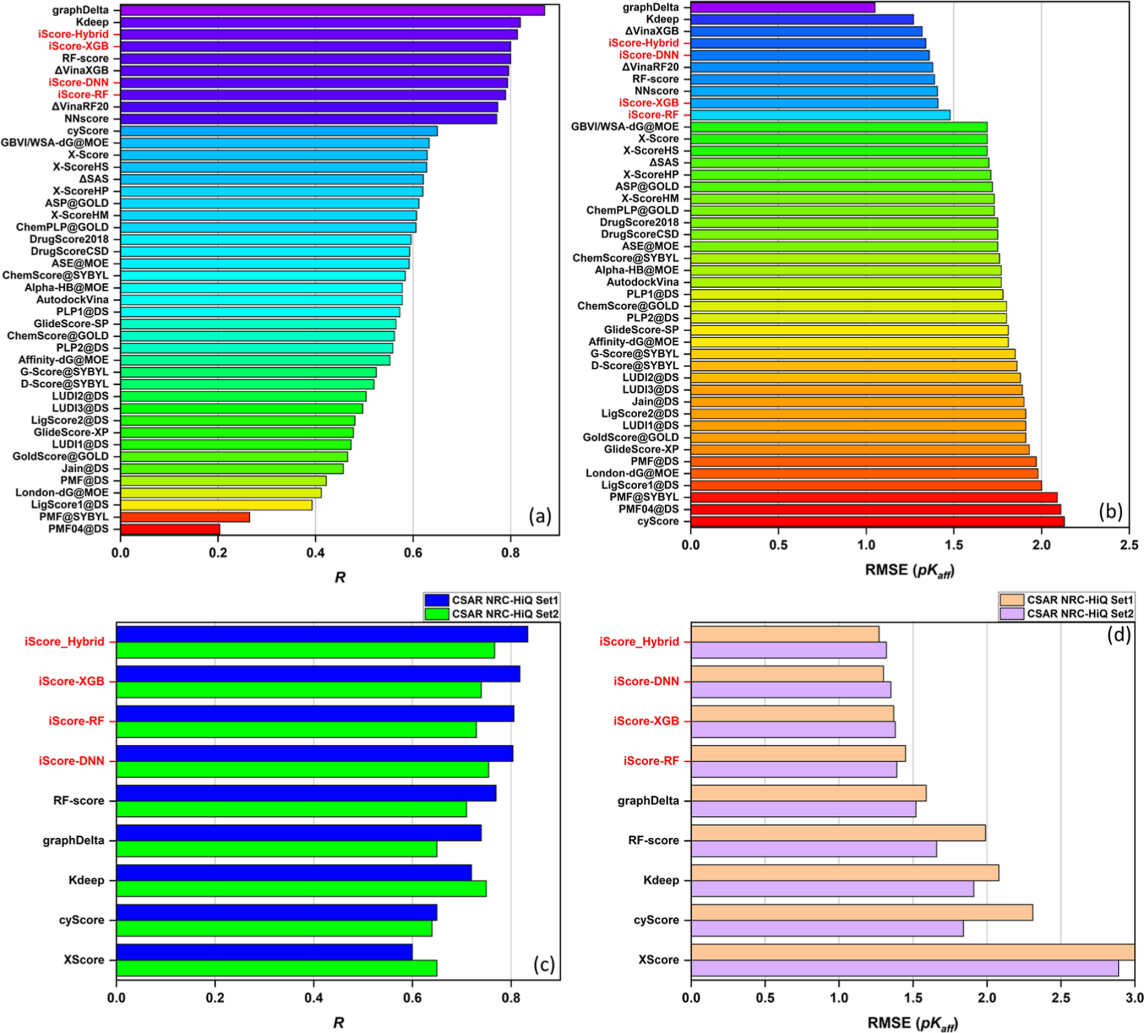
Scoring power performance of iScore models compared to other scoring functions tested on the (a,b) PDBbind 2016 core set and (c,d) CSAR NRC-HiQ Set1 and Set2. Scoring functions are ranked by the Pearson correlation coefficients in descending order.

Scatter plots comparing the predicted to the experimental values on the PDBbind-2016 core set are shown in Figure S4e–h. The 99% prediction band (pink area), Pearson correlation coefficient (*R*), and RMSE values are shown for each correlation. Figure 4a,b illustrates the scoring power performance of the iScore models versus 40 traditional and modern ML-based scoring functions in terms of the Pearson correlation coefficient and RMSE metrics tested on the PDBbind 2016 core set, respectively. As these figures show, iScore-Hybrid outperforms the base learners in the context of scoring power metrics (*R* = 0.814 and RMSE = 1.30) and ranks among the top scoring functions. Two major competitors are graphDelta^37^ and K_deep_.^38^ graphDelta is a ML graph-based scoring function that employs a message-passing neural network for modeling protein–ligand interactions and yielded the best scoring power metrics on the PDBbind 2016 core set with *R* = 0.87 and RMSE = 1.05. *K*_deep_ is also an ML-based scoring function that uses a 3D-convolutional neural network for predicting the ligand binding affinities. *K*_deep_ demonstrated a very good scoring power performance on the PDBbind 2016 core set with *R* = 0.82 and RMSE = 1.27. Nonetheless, these scoring functions, like any other scoring functions published up to now, require a full picture of the protein–ligand interactions, which imposes some critical limitations on their speed and applicability as discussed in the Introduction. Figure 4c,d compares the Pearson correlation coefficient and RMSE metrics of the iScore models against modern ML-based scoring functions, tested on CSAR NRC-HiQ Set1and Set2, respectively. iScore-Hybrid is among the top 3 best performing scoring functions on the PDBbind 2016 core set and is at the top of the list once tested on CSAR NRC-HiQ Set1 (*R* = 0.834 and RMSE = 1.27) and CSAR NRC-HiQ Set2 (*R* = 0.767 and RMSE = 1.32). The iScore base learners also outperform the other scoring functions including graphDelta (*R* = 0.74, 0.65 and RMSE = 1.59, 1.52) and *K*_deep_ (*R* = 0.72, 0.75 and RMSE = 2.08, 1.91) in these sets. As the test results indicate (Figure 4), iScore-XGB and iScore-DNN are the best performing base learners in terms of Pearson correlation coefficient and RMSE metrics on all three test sets, respectively.

**Figure 5:**
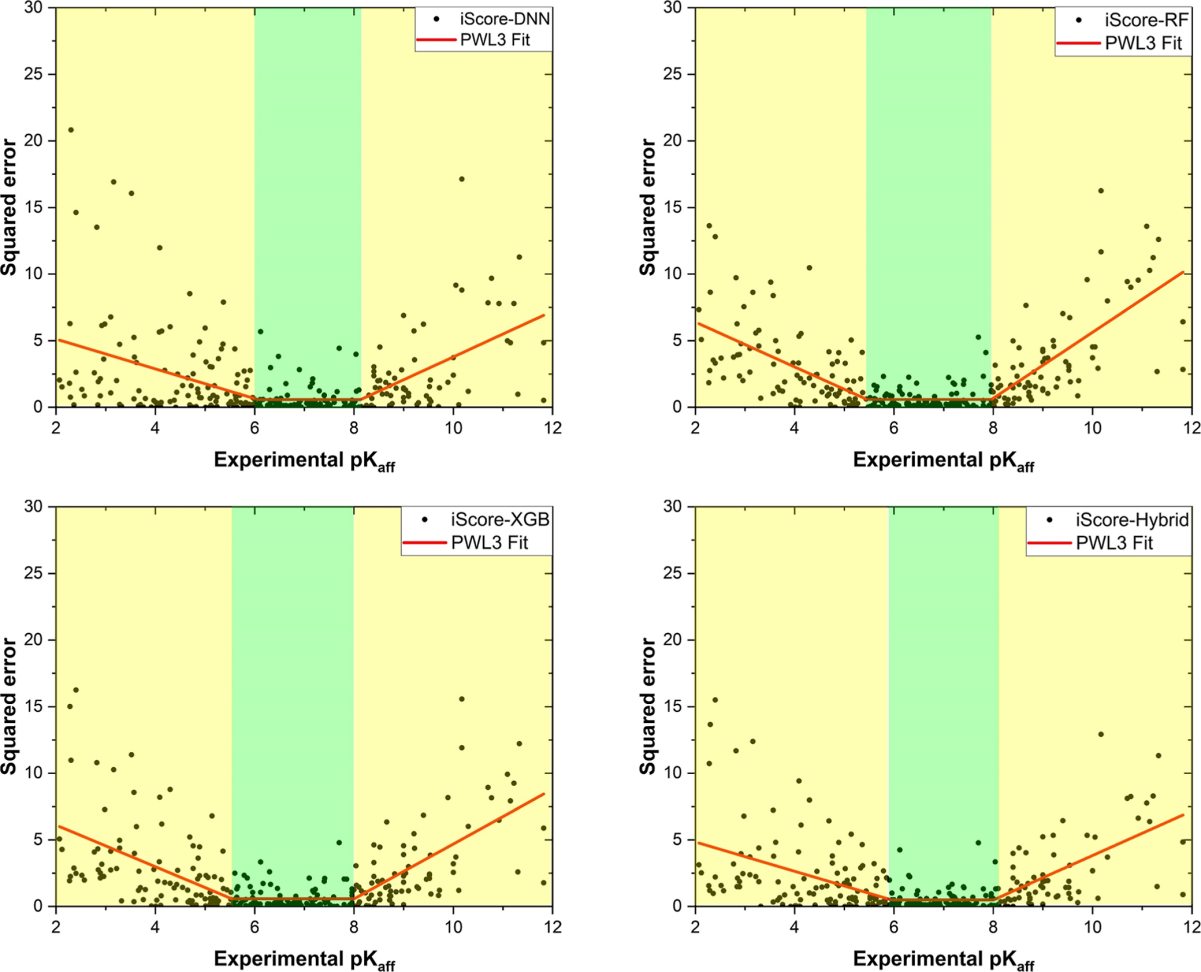
Squared error versus the experimental *pK*_d_ obtained from each model upon testing campaign on the PDBbind 2016 core set. The piecewise linear function with three segments (PWL3) fitted into the data is shown in red. The green and yellow dashed areas illustrate the trust zone and nonzero-slope regions, respectively.

Figure 5 illustrates the squared error (squared difference between experimental and predicted *pK*_d_) versus the experimental *pK*_d_ associated with each iScore model obtained on the PDBbind 2016 core set. The trust zone’s lengths of iScore-Hybrid and iScore-DNN are similar (∼2.1 [6.0, 8.1]), while iScore-RF and iScore-XGB show a higher value (∼2.5 [5.5, 8.0]). iScore-Hybrid has the lowest trust zone’s height, 0.48, followed by 0.56 (iScore-DNN), 0.59 (iScore-RF), and 0.60 (iScore-XGB). Furthermore, iScore-Hybrid shows the best performance at the low- and high-affinity limits where the absolute slope of the lines in these regions is 1.10 and 1.67, respectively. The corresponding values are (1.12 and 1.72), (1.68 and 2.48), and (1.56 and 2.06), for iScore-DNN, iScore-RF, and iScore-XGB, respectively. Hence, except for the trust zone’s length, iScore-Hybrid outperforms the base learners.

To demonstrate that the models effectively capture protein–ligand interactions, an ablation study was conducted by training two separate models for each base learner—DNN, RF, and XGB. One model utilized only ligand descriptors, while the other relied solely on binding pocket descriptors. The scoring power of these ablation models was then evaluated on the CASF-2016 data set. As shown in Figure S5, the results clearly indicate that using information from only one source (either the ligand or binding pocket) is insufficient for training robust models.

#### 3.2.2 Ranking Power

**Figure 6:**
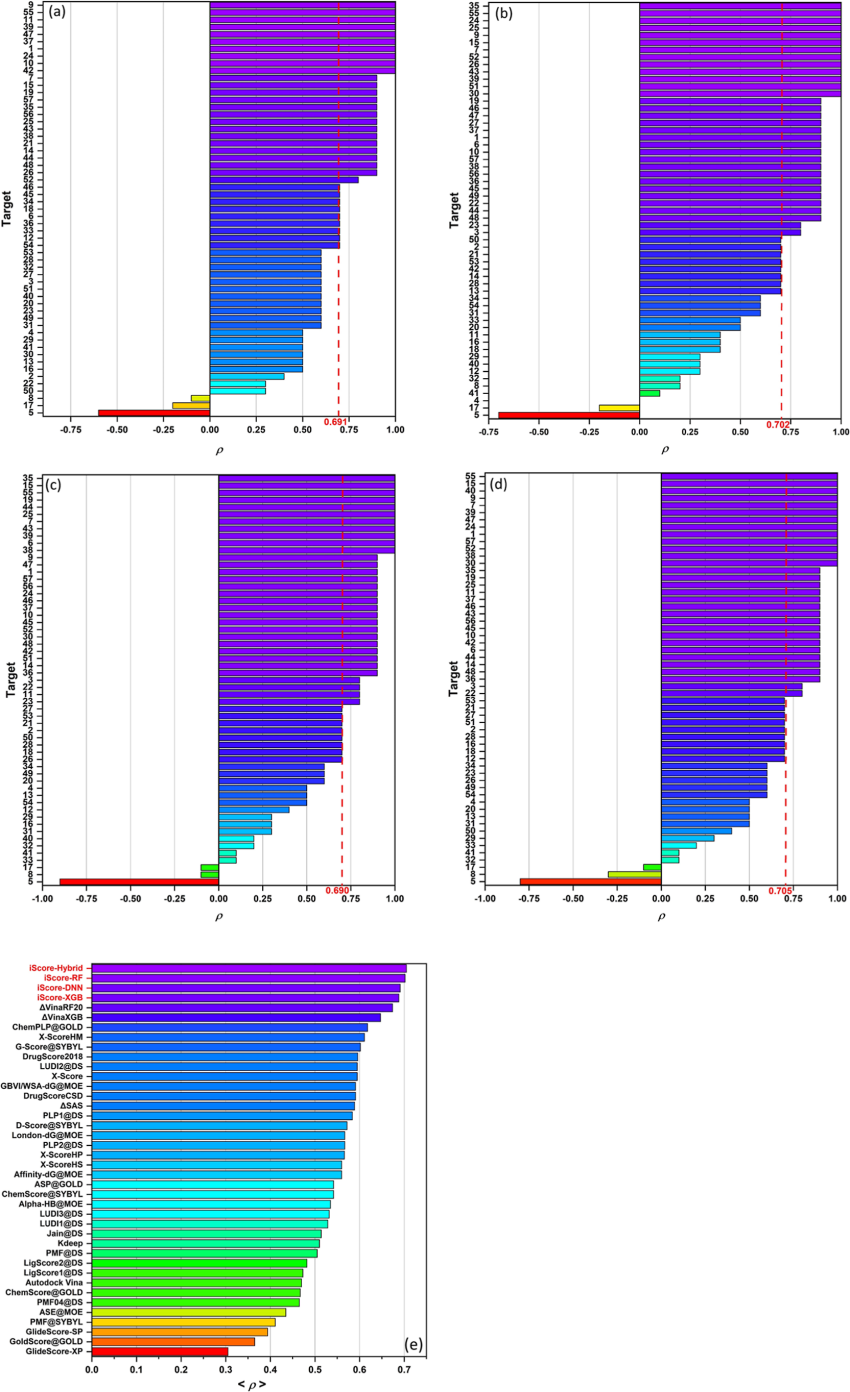
(a) Ranking power performance of (a) iScore-DNN, (b) iScore-RF, (c)-iScore-XGB, and (d) iScore-Hybrid on the CASF-2016 test set based on the Spearman correlation coefficient of individual targets. (e) Comparison between the ranking power performance of iScore models and other scoring functions based on the average Spearman correlation coefficients (vertical red dashed lines in (a–d)) over all targets.

Figure 6a–d shows the per-target and average (vertical red dashed lines) ranking Spearman correlation coefficients (ρ) over 57 targets in the PDBbind 2016 core set evaluated by the iScore base learners and the hybrid model. Figure 6e illustrates the ranking power performance of the iScore models (based on the average Spearman correlation coefficient) and compares those with several other scoring functions. As this figure shows, iScore-Hybrid (<ρ> = 0.705) outperforms not only the base learners but all other scoring functions in the ranking campaign. As Figure 6e indicates, iScore-Hybrid is followed by iScore-RF (<ρ> = 0.702), iScore-DNN (<ρ> = 0.691), and iScore-XGB (<ρ> = 0.690), respectively. It is worth to mention that the ranking power performances of iScore models are significantly better than *K*_deep_ (<ρ> = 0.51) which was one of the major competitors when comparing scoring power.

#### 3.2.3 Screening Power

**Figure 7:**
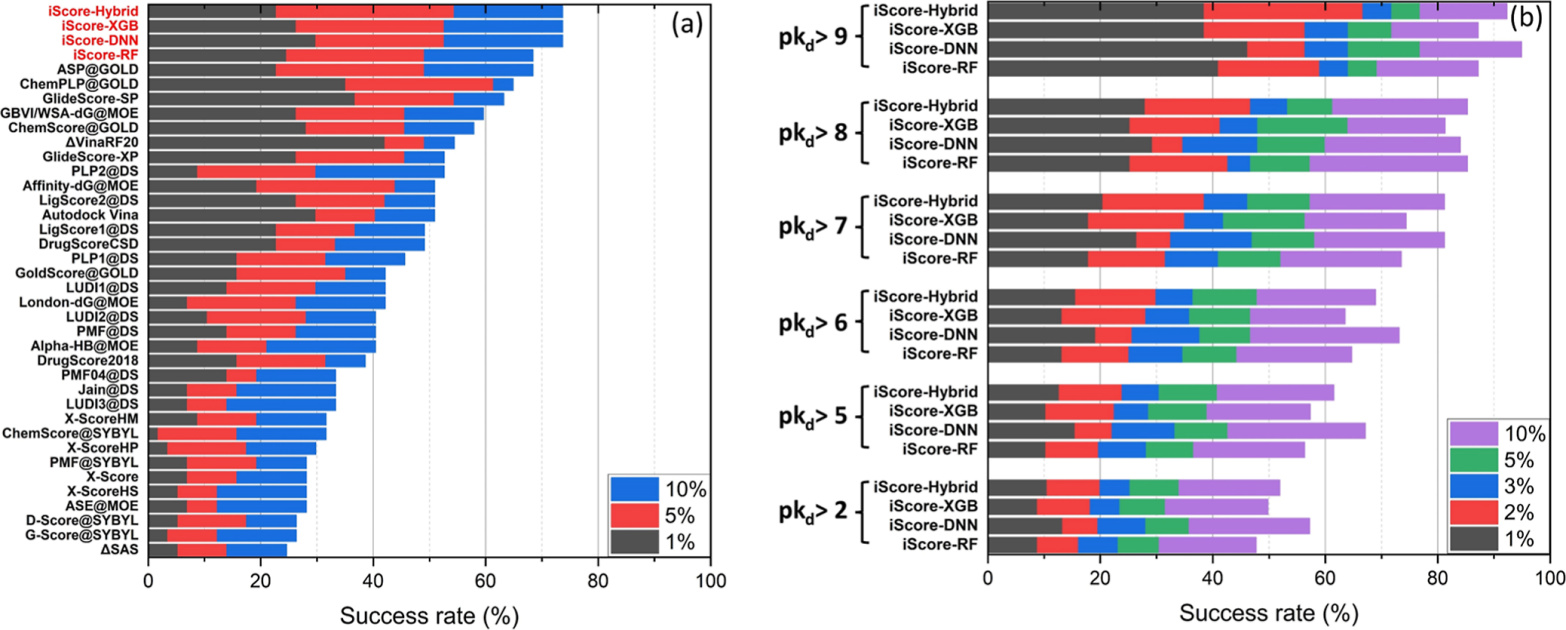
(a) Comparison between screening power performance of iScore and other scoring functions in the success rate of identifying the highest affinity ligand of each 57 target receptors, in the PDBbind 2016 core set, among the 1%, 5%, and 10% top candidates. (b) Success rate of identifying all binders with the experimental binding affinity values less than 10 mM (*pK*_d_ ≥ 2), 10 μM (*pK*_d_ ≥ 5), 1 μM (*pK*_d_ ≥ 6), 0.1 μM (*pK*_d_ ≥ 7), 0.01 μM (*pK*_d_ ≥ 8), and 1 nM (*pK*_d_ ≥ 9), among the 1%, 2%, 3%, 5%, and 10% top candidates over all 285 complexes in the PDBbind core set.

Figure 7a shows the screening power performance of iScore in terms of the success rate of identifying the highest affinity ligand of each 57 target receptors, in the PDBbind 2016 core set, among the 1%, 5%, and 10% top candidates. Figure 7a demonstrates that the screening performance of iScore (73.7% for iScore-Hybrid, iScore-XGB, and iScore-DNN and 68.4% for iScore-RF) is considerably better than that of all other scoring functions in the screening power campaign. Figure 7b illustrates the success rate of identifying all binders with experimental binding affinity values less than 10 mM (*pK*_d_ ≥ 2), 10 μM (*pK*_d_ ≥ 5), 1 μM (*pK*_d_ ≥ 6), 0.1 μM (*pK*_d_ ≥ 7), 0.01 μM (*pK*_d_ ≥ 8), and 1 nM (*pK*_d_ ≥ 9), among the 1%, 2%, 3%, 5%, and 10% top candidates over all 285 complexes in the PDBbind core set. As this figure shows, the success rate of iScore increases as *pK*_d_ of the binder increases. For instance, the success rate of iScore-Hybrid is 51.9, 61.5, 68.9, 81.2, 85.3, and 92.3% for identifying all binders with the experimental binding affinity values less than 10 mM, 10 μM, 1 μM, 0.1 μM, 0.01 μM, and 1 nM, among the 10% top candidates.

For a more robust evaluation, the screening power of iScore was further benchmarked against the DUD-E data set.^22^ Due to the hidden bias observed in this data set,^39−41^ it is not recommended for use as a training set in ML classification tasks, as it may misleadingly yield strong internal validation results but poor generalization to unseen data. Nonetheless, DUD-E remains a valid benchmark for evaluating the screening power of iScore. As mentioned earlier, since the actives and decoys in this data set share similar 1D descriptors, iScore’s performance in this case must rely solely on differences in 2D descriptors.

**Figure 8:**
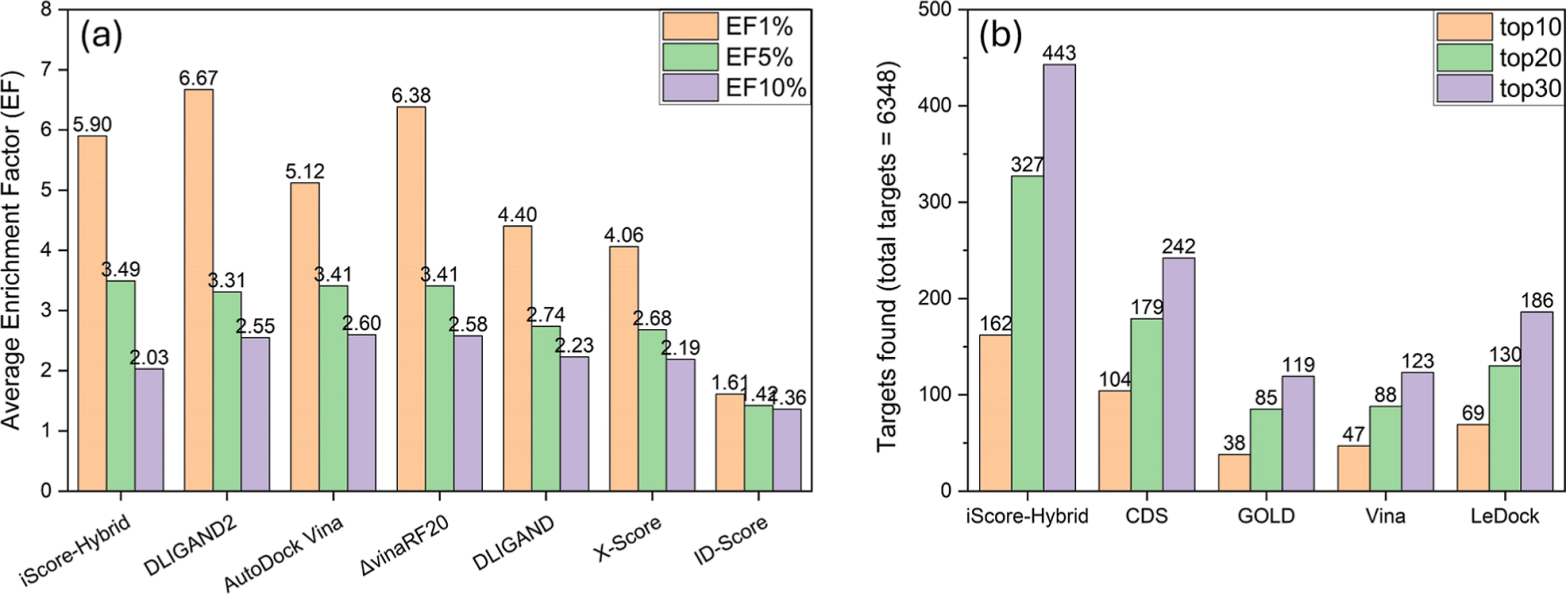
(a) Comparison of iScore-Hybrid’s screening power performance with six other scoring functions, evaluated using the average EFs at the top 1% (EF1%), top 5% (EF5%), and top 10% (EF10%) on the DUD-E benchmark. (b) Comparison of iScore-Hybrid’s target fishing performance against four different scoring functions, assessed based on the total number of targets identified within the top 10%, 20%, and 30%.

The DUD-E data set was downloaded and prepared following the approach described in Materials and Methods. The prepared structures of protein–ligand complexes, and active and decoy ligands in SMILES format, are freely available in the Zenodo repository at (DOI: 10.5281/zenodo.14865257). The ligand descriptors and protein binding pocket features were calculated by using RDKit and FPocket, respectively. Subsequently, the binding affinity of each target protein against its active and decoys was predicted by the iScore models and sorted. The Enrichment Factor (EF) at the top 1% (EF1%), 5% (EF5%), and 10% (EF10%) was calculated using the following equation
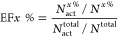
5where *N*_act_^*x* %^, *N*^*x* %^, *N*_act_^total^, and *N*^total^ are the number of actives found in the top *x* %, number of compounds (actives and decoys) in the top *x* %, total number of actives per target protein, and total number of compounds (actives and decoys) per target protein, respectively. Figure S6 shows EF1%, EF5%, and EF10% per target protein in the DUD-E data set, with the average EFs presented in Figure 8a and compared to six scoring functions reported by Chen et al.^42^ The results indicate that iScore ranked third in terms of EF1% (5.90), following DLIGAND2 (6.67) and ΔvinaRF20 (6.38). However, iScore outperforms the other scoring functions in terms of EF5% (3.49) and is the second lowest EF10% (2.03) after ID-Score (1.36). While these benchmarking results clearly demonstrate that 2D molecular descriptors can be used to distinguish actives from decoys in the DUD-E data set, the importance of 1D molecular descriptors for the iScore model in making an effective classification remains undeniable.

#### 3.2.4 Target Fishing

To verify that the iScore models genuinely utilize binding pocket information in affinity predictions—rather than making spurious correlations with features specific to the current training and test sets—a target fishing (reverse docking) study was conducted, as outlined by Lee and Kim.^23^ The objective of reverse docking is to identify true targets among a diverse set of clinically relevant protein targets.

The drug molecules and target proteins were retrieved from DrugBank (http://www.drugbank.ca) and PDB (https://www.rcsb.org/), respectively, and prepared following the approach described in Material and Methods. The prepared structures of protein–ligand complexes and prepared drug molecules in SDF format are freely available in the Zenodo repository at DOI: 10.5281/zenodo.14865257. Table S3 lists the drug names, their associated PubChem- and DrugBank-id’s, PDB-id, and number of active target proteins per drug molecule. The cocrystallized ligands were used to define the protein binding pocket for its descriptor calculations. The molecular descriptors for each drug were calculated using the RDKit tool. Subsequently, the binding affinity for each drug against all 1860 unique target proteins was predicted by the iScore models and sorted. Figures S7 and 8b present the per-drug and total number of targets identified within the top 10%, 20%, and 30%, respectively. As illustrated in Figure 8b, iScore-Hybrid significantly outperforms the other scoring functions. The total number of active targets identified by iScore-Hybrid at the top 10%, 20%, and 30% thresholds is 162, 327, and 443, respectively, whereas the second-best scoring function “Consensus Docking Score”, identified only 104, 179, and 242 active targets at the same thresholds.

### 3.3 Speed Performance

The iScore models were trained using a single compute node on the Alvis supercomputer allocated by the C3SE supercomputing facility, with one NVIDIA Tesla A100 HGX GPU (40GB RAM), 32 core Intel(R) Xeon(R) Gold 6338 CPU 2 GHz, and 256GB DDR4 RAM. iScore is capable of screening >8000 compound/s (∼700 million screenings a day) on a single compute node: 32 cores AMD Ryzen 9 7950X CPU, NVIDIA RTX A4000 GPU (16GB RAM), and 32GB DDR5 RAM.

## 4 Conclusions

This work introduces iScore, a cutting-edge ML-based scoring function designed to predict the binding affinity of protein–ligand complexes with unprecedented precision and speed. Unlike traditional scoring functions that rely heavily on the explicit knowledge of intermolecular interactions obtained from explicit binding poses, iScore leverages a novel approach. It utilizes a combination of ligand and binding pocket descriptors to directly predict binding affinities, thereby bypassing the need for extensive conformational sampling. This methodological innovation not only saves significant computational time and resources but also provides the applicability to evaluate vast molecular libraries, offering a leap toward exploring the chemical space more efficiently. The benchmarking of iScore across multiple data sets highlights its robustness and superior performance over traditional and advanced scoring functions. Notably, the development of the hybrid iScore model (iScore-Hybrid), which integrates the strengths of individual base learners, sets new benchmarks in scoring, ranking, screening, and target fishing capabilities, which are essential for drug discovery processes. The innovation of iScore is further underscored by its practical implications. The ability to screen over 8000 compounds per second on a single GPU translates to the screening of 700 million compounds daily, illustrating the scalability and efficiency of iScore in handling ultralarge molecular libraries. This capability is critical in accelerating the drug discovery process, from initial screening to identification of lead compounds. The promising results of iScore not only benchmark a new standard in scoring function development but also open a new era in the utilization of ML technologies for drug discovery.

## Data Availability

The training data sets, pretrained models, and instruction for the retraining is available at https://github.com/i-TripleD/iScore. The prepared DUD-E and target fishing data sets are freely available in the Zenodo repository at zeonodo.org, with DOI: 10.5281/zenodo.14865257.

## Supporting Information Available

Additional information regarding the pdb codes used for training and benchmarking, database analysis, molecular and binding pocket descriptors, training and test scatter plots, per-target EFs for DUD-E benchmark, and per-drug active targets found for target fishing benchmark (PDF)
